# Translation, Validity, and Reliability of a Persian Version of the Iowa Tinnitus Handicap Questionnaire

**Published:** 2014-04

**Authors:** Homa Arian Nahad, Masomeh Rouzbahani, Farnoush Jarollahi, Shohreh Jalaie, Akram Pourbakht, Helnaz Mokrian, Parvane Mahdi, Amin Amali, Abdolmajid Nodin Zadeh

**Affiliations:** 1 *Department of Audiology, School of Rehabilitation Sciences, Iran University of Medical Sciences, Tehran, Iran.*; 2 *Department of Statistics, School of Rehabilitation, Tehran University of Medical Sciences, Tehran, Iran.*; 3 *Occupational Sleep Research Center, Department of Otorhinolaryngology, Imam Khomeini Educational Complex Hospital, Valiasr Hospital,* * Tehran University of Medical Sciences, Tehran, Iran.*; 4 *Department of Architect Engineering, Schoolof Technology And Engineering. Islamic Azad University of Bandar Abbas.*

**Keywords:** Tinnitus Handicap Questionnaire, Translation, Reliability, Validity, Persian.

## Abstract

**Introduction:**

Tinnitus is a common otologic symptom that can seriously affect a patient’s quality of life. The purpose of the present study was to translate and validate the Iowa Tinnitus Handicap Questionnaire (THQ) into the Persian language, and to make it applicable as a tool for determining the effects of tinnitus on a patient’s life.

**Materials and Methods:**

The main version of the THQ was translated into the Persian language. The agreed Persian version was administered to 150 tinnitus patients. The validity of the Persian THQ was evaluated and internal reliability was confirmed using Cronbach’s α-coefficient. Finally, the effect of independent variables such as age, mean patient threshold, gender, and duration of tinnitus were considered in order to determine the psychometric properties of tinnitus.

**Results:**

After an exact translation process, the Persian THQ was found to exhibit face validity. In terms of content validity, content validity index in total questionnaire was 0.93. Further, in structural validity measurements, intermediate correlation with annoyance from tinnitus (r=0.49), low correlation with duration of tinnitus (r=0.34) and high correlation with the Tinnitus Handicap Inventory (THI) questionnaire (r=0.84) were demonstrated. Additionally, a negligible effect of gender and age was noted on degree of tinnitus handicap (P= 0.754, P= 0.573, respectively). In the internal reliability assessment for Factors 1, 2, 3, and the whole questionnaire, Cronbach`s α-coefficient was 0.95, 0.92, 0.25 and 0.88, respectively.

**Conclusion:**

The Persian version of the Iowa THQ demonstrates high validity and reliability and can be used for the determination of tinnitus handicap and for following-up in the intervention process in Persian tinnitus patients.

## Introduction

Tinnitus is a complaint of repeated sound that is perceived in one or both ears or in the whole of the head (1). It is a prevalent symptom that is usually accompanied by sensory and neural hearing loss; however it may also occur spontaneously (2). The chronic condition is reported in 5–10% of adults. Most patients adapt to this phantom perception of sound; however in 20% of the sufferers who fail to adapt, tinnitus may lead to disturbances in the patient’s life in the form of agitation, depression, fear, sedation, disturbance, lack of concentration, and sleep difficulties (3). According to published reports, tinnitus has led to suicide in 2–4% of cases (4). Stephens et al (1994) reported the presence of tinnitus in 70% of patients with depression and in 18% of people who attempt suicide unsuccessfully (5). 

Distress, tension disorders, psychological disorders and difficulties related to sleep, and disturbances in daily activities tend to mandate remediation (6).

In general, the relationship between tinnitus features or psychoacoustic characteristics, such as pitch and loudness, and the perceived severity of tinnitus and its emotional impact are inconsistent. Therefore, the application of self-reported scales in tinnitus patients is essential, as it provides supplementary information to conventional tinnitus assessments (1).

One of the most important psychometric assessment tools in the determination of the effects of tinnitus on quality of life is a self-reported handicap questionnaire (7). 

To date, many questionnaires have been established for this kind of assessment, including the Tinnitus Effect Questionnaire (8), Tinnitus Severity Scale (9), Subjective Tinnitus Severity Scale (10), Tinnitus Handicap Inventory (THI) (11), Tinnitus Reaction Questionnaire (10), and Tinnitus Handicap/Support Scale (12). As most of the questionnaires assessing the effects of tinnitus on quality of life are in the English language, the provision of a Persian version, consistent with Iranian culture, is opportune. The first tinnitus questionnaire translated into the Persian language was the Tinnitus Handicap Inventory (THI), which was standardized in 112 people by Mahmoudian et al (2011) and obtained high internal consistency and reliability (Cronbach’s α-coefficient = 94%)    (13) . The main version of the THI questionnaire was first developed by Newman et al in 1996, with the purpose of assessing the functional, emotional, and catastrophic effects of tinnitus (11). The Persian version of this questionnaire was completed in all of our patients and was scored on a 3-point scale, in which participants were required to select between “Yes, “No”, and “Occasionally” (17). The relevant score for these choices were 4, 0, and 2, respectively    (13).

The Tinnitus Handicap Questionnaire (THQ) was first developed by Kuk et al in Iowa University in 1990. It has 27 items and includes three factors. In Factor 1, Questions 5,6,9,12, and 13 assess the behavioral effects of tinnitus; Questions 19, 18, 10, and 23 assess social effects; and Questions 14, 20, 21, 25, 26, and 27 assess emotional effects. In Factor 2, Questions 2,1,4,7,17,16,24, and 22 assess hearing abilities of tinnitus patients, while Factor 3 includes Questions 3,8,15, and 11 that assess the patient’s perspective    (14) . 

This questionnaire is useful for objectifying the impact of tinnitus on the patient’s life, and provides data that determine the handicap arising from tinnitus. Additionally, it makes it possible to compare the consequence of tinnitus between patients and also provides a framework for management and treatment processes        (14,15) . This questionnaire evaluates large areas of tinnitus affection, and, by using wide scale (ranging from 0 to 100) allows better sensitivity and the potential to detect even small changes in the treatment and assessment process        (16). In this study, the THQ was translated and standardized into the Persian language and then its validity and reliability were evaluated. The study was conducted according to the International Quality Of Life Assessment (IQOLA) protocol that was approved by World Health Organization (WHO) in 1991. The aim of this protocol was to generate a health questionnaire with high validity and reliability, and to make it applicable in different countries according to their formal language. 

## Materials and Methods

*Translation of the Iowa THQ into the Persian language:*

We translated the main version of the Iowa THQ according to the IQOLA protocol, during a 3-month period from July to October 2012. The translation process was conducted in two phases. In the first phase, forward translation was undertaken by two translators (Translators 1 and 2) who had sufficient proficiency in the English and Persian languages and who were experienced in questionnaire translating. The translators were asked to prepare a list of alternative translations for some words, if required. Then, each of the translators were asked about the difficulties of translation and gave a score according to a 100-point Visual Analogue Scale (VAS), from zero (easy translation) to 100 (difficult translation). An average score of more than 30 was considered a difficult translation. The agreed Persian version of the questionnaire was then submitted to two other translators who were skilled in Persian philology (Translators 3 and 4), for determination of translation quality and to provide the quality score. The quality of translation included the clarity of the text, conceptual equivalence (similarity of content/ meaning), and usage of common language, in order to be an acceptable Persian version. In the second phase of translation, the back translation phase, two English native translators (Translators 5 and 6) were asked to translate the approved Persian version of the questionnaire into the English language. Finally, we compared the translations of these two translators with the translation of the main version of the questionnaire, as performed by the previous translators.

*Measurement of questionnaire validity and reliability:*

In this phase, in order to confirm the validity of the Persian version of the questionnaire, an audiologist as well as an audiologist and a group of tinnitus sufferers determined the validity through the use of descriptive scales. In order to determine the content validity of the questionnaire, a 3-point scale was assigned to all items and the Content Validity Ratio (CVR) was computed (17). Face validity was measured using aspects of fluency (using meaningful words) and cultural acceptance in society, according to a 6-point scale (very weak, weak, moderate, good, very good, best). There are many methods available to determine the structural validity of a questionnaire in tinnitus, including evaluating tinnitus handicap, as annoyance is one aspect of a psychometric handicap of tinnitus. The Spearman product-moment correlations between the extent of the patient’s annoyance (zero to 100-point scale on subjective reported annoyance) and the mean score of the Persian version of the THQ were measured for this purpose. In the next step, for measurement of convergent validity, we applied the Persian version of the THI questionnaire. To prevent an order effect, the order of questionnaire filling was changed across all patients. Approximately 150 patients completed the initial questionnaire using a paper-and-pencil format at the time of their audiological evaluation. In order to determine the reliability, Cronbach’s α-coefficient was used, while additionally approximately one-third of our patients (n=40) participated in a second adminis- tration of the questionnaire 3–7 days later, in order to provide data to determine reliability across two administrations. Then, the scores of each factor and the total score were computed for calculation of the repeated coefficient, the intra-class correlation coefficient (ICC). Finally, the effect of independent variables such as age, duration, and extent of annoyance were calculated using the correlation coefficient. One-way analysis of variance and an independent t-test were applied to determine the effect of patients’ mean threshold (in dB HL) from 250 to 8000 Hz at octave intervals and sex, respectively.

*Subjects*

The final Persian version of the Iowa THQ questionnaire was applied to 150 tinnitus sufferers, all of whom attended an otorhinolaryngology clinic at Amir Alam Educational Hospital, Tehran University of Medical Sciences. The mean (SD) age of patients was 49.32 (14.96) years (range, 19–88 years); 93 (62%) patients had unilateral and 39 (26%) patients had bilateral tinnitus, while 18 (12%) stated their tinnitus perception was in the whole of the head. Among the patients, 36 (24%) had normal hearing and 114 (76%) had varying levels of hearing loss. The median duration of the tinnitus complaint was 45.86 months (range, 3–360 months). All patients were visited by an ear, nose and throat (ENT) specialist (author 8) for the exclusion of any otologic abnormalities such as ear discharge and cerumen impaction, for example. The following inclusion criteria for patients were applied during participants’ selection: 1) age >18 years, 2) history of tinnitus for >3 months, and 3) air bone gap (ABG) ≤5 dB. After obtaining informed consent and local ethics committee approval, all participants underwent otoscopy, tympanometry, and audiometry tests. Finally, the Persian version of the Iowa THQ was completed in all subjects. 

*Statistical analysis*

 SPSS software, version 11.5 (Chicago, IL, USA), was used for statistical evaluation. In order to determine the construct validity, the non-parametric test, Spearman correlation coefficient, was used. The parametric test, Pearson correlation coefficient, was used for calculating the convergent validity. For evaluation of the internal reliability of the Persian version of the Iowa THQ and its retest, Cronbach’s α-coefficient ICC, and an independent Student t-test and Pearson correlation coefficient were used. The effects of gender and auditory thresholds on the questionnaire scores were assessed using an independent t-test and a one-way analysis of variance (ANOVA). In all statistical procedures, a P-value <0.05 was considered statistically significant.

## Results

The patient group consisted of 74 (49.33%) females, with a mean (SD) age of 49.20 (14.18) years and 76 (50.66%) males, with mean (SD) age of 49.43 (15.83) years. The mean (SD) of total score, score of Factor 1, Factor 2, and Factor 3 was 35.05 (23.40), 32.66 (25.21), 35.28 (26.48), and 43.28 (21.7), respectively. There were no statistically significant differences between tinnitus sufferers with respect to age (P=0.573). 

In the Persian version of the THQ, gender as an independent variable did not affect the degree of handicap perceived by the patients. From a statistical point of view, using an independent t-test, there were no significant differences between the two genders in total scores (P=0.754). Data relating to gender effect on the mean score of questionnaire are presented in Table 1, with details of each factor. 

*Translating and developing Persian version of Iowa THQ:*

In order to generate the agreed Persian version of the questionnaire, the translators gave a difficulty score, based on the visual score (0–100), for each of the 27 items. All questions obtained a simple and relatively simple translation difficulty quality, with the exception of Questions 3, 8, 17, and 18. According to translation quality criteria, those questions which did not achieve a desirable score in the fields of clarity, common language, conceptual equivalence, 

and total quality of translation, were revised accordingly. Finally, all questions in the agreed Persian version of the questionnaire obtained a score >90 and were considered to be of appropriate quality. Table 2 illustrates the main version of the Iowa THQ.

**Table 1:** Mean score on each subscale of the tinnitus questionnaire in males and females

		**Male ( n= 76)**		**Female (n = 74)**		**Female (n = 74)**	
	P -value	Mean	SD	mean	SD	mean	SD
Factor 1	0.835	31/01	24/97	34/35	25/51	32/66	25/21
Factor 2	0.631	35/6	26/84	35/20	26/29	35/28	26/48
Factor 3	0.369	41/90	2139	44/70	20/78	43/28	21/07
total core	0.754	34/14	23/29	36/12	23/55	35/05	23/40

**Table 2:** Main version of the Iowa THQ

I am unable to follow a conversation during meetings because of tinnitus.
Tinnitus creates family problems.
I think I have a healthy outlook on tinnitus.
I feel uneasy in social situations because of tinnitus
I have trouble falling asleep at night because of tinnitus.
Tinnitus contributes to a feeling of general ill health.
Tinnitus interferes with my ability to tell where sounds are coming from.
I have support from my friends regarding my tinnitus.
I am unable to relax because of tinnitus.
I do not enjoy life because of tinnitus.
My tinnitus has gotten worse over the years.
I cannot concentrate because of tinnitus.
Tinnitus makes me feel tired.
Tinnitus causes me to feel depressed
The general public does not know about the devastating nature of tinnitus.
Tinnitus causes me to avoid noisy situations.
Tinnitus interferes with my speech understanding when talking with someone in a noisy room
I find it difficult to explain what tinnitus is to others.
I complain more because of tinnitus
Tinnitus makes me feel annoyed.
Tinnitus makes me feel insecure
Tinnitus interferes with my speech understanding when listening to the television
Tinnitus affects the quality of my relationships.
Tinnitus has caused a reduction in my speech understanding ability.
Tinnitus causes stress.
Tinnitus makes me feel anxious.
I feel frustrated frequently because of tinnitus.

*Questionnaire validity*

In the evaluation of questionnaire face validity, all 27 items obtained a score >4 in the 6-point descriptive scale in 80% of participators; however, Questions 3, 8, 15, and 18 achieved a marginal score. From the point of content validity, among 27 items, all CVR scores were acceptable, and CVI in total questionnaire was 0.93.

 As shown in Table 3, Factors 1and 2 had a Pearson correlation coefficient of 0.89, Factors 1 and 3 had a Pearson correlation coefficient of 0.62, and the corresponding value for Factors 2 and 3 was 0.60.

The Pearson correlation between age and total score (Table 4) was 0.046 (P=0.573), indicating that age had no significant effect on extent of handicap in patients. 

 In the one-way ANOVA test, patients’ mean thresholds (in dB HL) indicated a full correlation (total score, r=0.16, P=0.040; Factor 2, r=0.23, P=0.002). As shown in Table 3, Factors 1 and 3 showed no meaningful correlation with hearing loss (P>0.05). 

**Table 3:** Correlations among the three factors

	**Factor 1**	**Factor 2**	**Factor 3**
Factor 1	1/00	0/89	0/62
Factor 2	0/85	1/00	0/60
Factor 3	0/62	0/60	1/00

**Table 4:** Correlation between score of questionnaire and the validation measures (n=150).

	**Total score THI**	**Annoyance**	**Mean Hearing Threshold**	**Duration**	**Age**
Factor 1	0.83* P = 0.000	0.57* P = 0.000	0.08 P = 0.152	0.36* P = 0.000	0.01 P = 0.409
					
Factor 2	0.80* P = 0.000	0.34* P = 0.000	0.23* P = 0.002	0.28* P = 0.000	0.10 P = 0.096
					
Factor 3	0.60* P = 0.000	0.32* P = 0.000	0.14 P = 0.077	0.27* P = 0.000	0.01 P = 0.880
					
Total score	0.84* P = 0.000	0.49* P = 0.000	0.16* P = 0.040	0.34* P = 0.000	0.04 P = 0.573

* Meaningful correlation

In structural validity measurements (Table 4), a moderate Spearman correlation coefficient was obtained in the annoyance interval scale (0 to 100; r = 0.49 and r = 0.57 for total score and Factor 1, respectively). Factors 2 and 3 had a low correlation with annoyance scale. Administration of the THI questionnaire (the only tinnitus questionnaire available as a Persian version) to determine convergent validity showed a high Pearson correlation with total score of the Persian version of the Iowa THQ (r= 0.84), Factor 1 (r= 0.83), and Factor 2 (r=0.80). 

**Fig 1:**
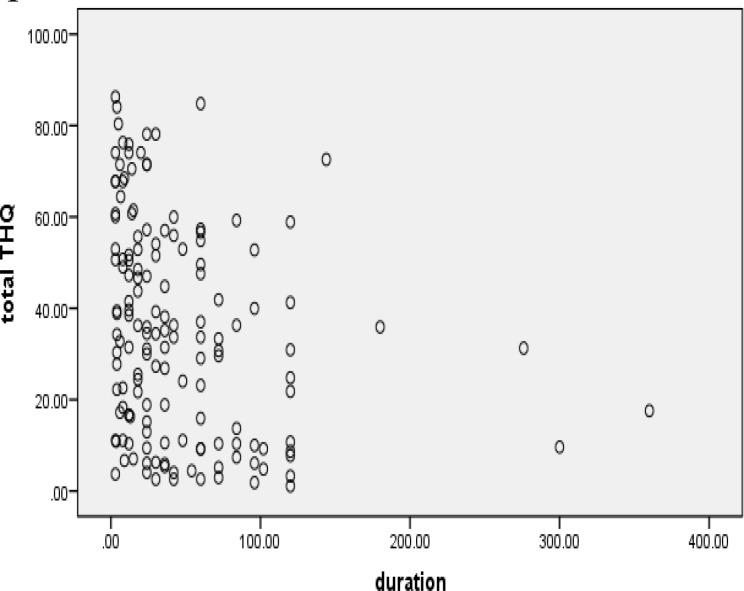
Dotted dispersion between duration of tinnitus and total score

The Spearman correlation coefficient between the duration of tinnitus and mean total score of the Persian version of the Iowa THQ was negative (r=−0.34, P=0.00) (Table 4). It was shown that in our patients, an increase in duration of tinnitus was not associated with a decrease in handicap, indicating an inverse relationship between these two parameters. Figure 1 shows the dotted dispersion of duration of tinnitus per total score. 

*Reliability of questionnaire*

**Fig 2:**
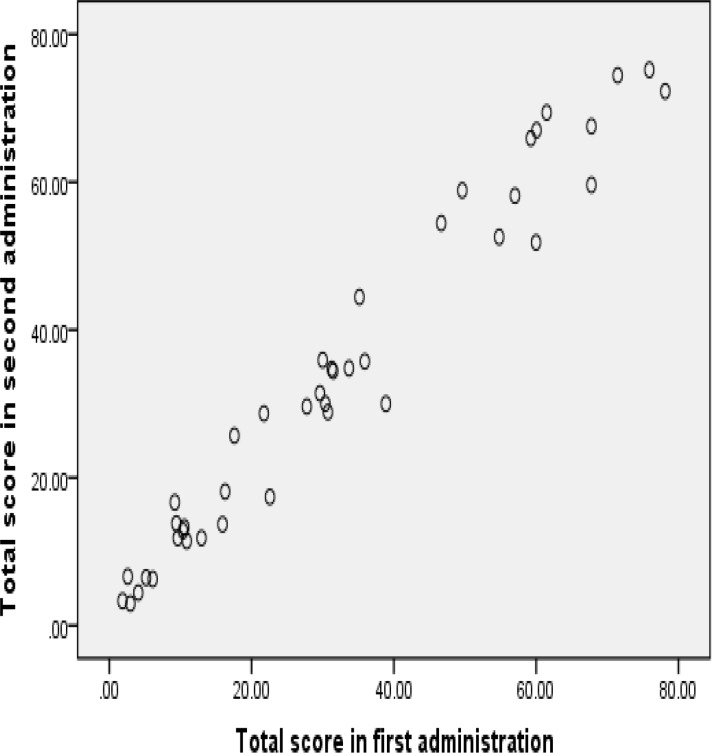
Dotted dispersion of total score across administrations (3–7 days apart)

In the assessment of the internal reliability of the Persian version of the Iowa THQ, the Cronbach α-coefficient for Factor 1, Factor 2, Factor 3, and the questionnaire with three factors were 0.95, 0.92, 0.25, and 0.88, respectively. In addition, the standard error of measurement (SEM) for the total score was ±8. In order to assess reliability, ICC was calculated in the first and second administration (3−7 days later) of the questionnaire. The ICC coefficient for total score was 0.98, and according to the independent t-test, there was no meaningful difference in the comparison of the mean total score between the first and second administration of the questionnaire (P=0.980). Figure 2 shows the dispersion of the total score of the Persian version of this questionnaire in the two administrations. Additionally, Pearson correlations between two administrations for Factors 1, 2, 3, and total score were 0.97, 0.95, 0.95, and 0.97 respectively. 

## Discussion

With three factors, the Persian version of this questionnaire, like the main version of the THQ, is suitable for the assessment and monitoring of areas of tinnitus handicap with high validity and reliability.

Factor 1 included 15 questions that assessed emotional, social, and physical aspects of tinnitus. Factor 2 included eight questions that evaluated hearing difficulties and Factor 3 included four questions that considered the patient’s perspective. Standardization of the Persian version of the questionnaire, like the main version, was performed across all 27 items. Therefore, for accurate comparison of each patient with normative data, all 27 items should be evaluated. In calculations involving the factors scores, as suggested by Kuk et al, the scores for items 3 and 8 should first be inverted (subtracted from 100) and then the degree of handicap be calculated (14).

After the exact translation process to the Persian language, with regard to the need for providing a self-report questionnaire for the assessment of the effect of tinnitus on a patients’ life and monitoring treatment outcomes in the Persian language, this questionnaire was administered in patients who had tinnitus. In order to prepare a questionnaire applicable in a target population, it is first necessary to obtain a desirable score for translation quality across aspects of clarity, common language, conceptual equivalence, and total quality of translation. Our questionnaire was based therefore based on this condition. 

In the first assessment of face validity undertaken by two groups (consisting of audiologists and a group of individuals suffering from tinnitus), all of the 27 items achieved the desired face validity (except Questions 3, 8, 15, and18). In terms of content validity, CVR scores among the 27 items were acceptable (except Questions 15 and 18).

In the structural validity measurement, as shown in Table 4, a moderate correlation coefficient was obtained between annoyance interval scales with total score of the Persian Iowa THQ. In the Dutch version of the THQ, moderate correlation has also been reported between two parameters. In fact, with medium correlation, especially between the total score and Factor 1, if the level of annoyance experienced by patients became greater, the mean degree of handicap arising from tinnitus was also greater  (4). Additionally, a low and inverse correlation between the duration of tinnitus and mean scores was obtained, indicating that as the duration of tinnitus increases, so the degree of handicap decreases due to adaptation.

In the convergent validity measurement of the Persian version of the THI, a high Pearson correlation with the Persian version of the THQ was observed. This is probably due to an area of handicap that affected the score in two the questionnaires.

Additionally, as shown in Table 4, a low correlation was noted between the mean hearing threshold with total score and Factor 1. This low correlation coefficient showed that despite difficulties of hearing, patients had little hearing handicap; in other words, they adapted to their hearing disorder successfully. In the main version of the questionnaire, there was no correlation between factors and mean hearing thresholds. This finding may be due to the difference in sample size between the Persian version (n=150) and the main version (n=45) (14).

 In the Persian version, Factor 1 had a high correlation with Factor 2. In comparison with the main version of the questionnaire devised by Kuk et al (r=0.49), a high correlation between Factor 1 and 2 indicates that in most tinnitus patients, a handicap in Factor 1 (handicap in social, emotional, or physical area) is accompanied by a handicap in Factor 2 (hearing difficulties) (14).

We obtained a high Cronbach α-coefficient in the assessment of the internal reliability of the questionnaire, in Factors 1, 2, and in the total questionnaire. Like the main version, low internal reliability was noted in Factor 3, indicating that this factor should not be used separately as an independent factor. The Persian version of the Iowa THQ obtained a high ICC coefficient across all three factors and total score. As reported by Nunnally et al(1978), a high correlation coefficient (≥80) between the two administrations is a criterion that means we can consider the questionnaire to be acceptable for clinical purposes (18). Pearson product-moment correlations were high between the two administrations in all three factors and total score. Test-retest stability of THQ was assessed by Newman for minimal contribution of memory; high correlation coefficients were obtained except for Factor 3. Additionally, the 3−7-day interval between administrations of the Persian version of the THQ indicated that, except for Factor 3, no other factors were affected by the short time interval between the two administrations (19). In our study, the total score of the Persian Iowa THQ and its first, second and third subscales were 35.05, 32.66, 35.28, and 43.28, respectively. The scores obtained were in accordance with the study of Kuk et al (1990) (14). In other words, the degree of handicap arising from tinnitus (regarding confidence intervals) was similar between these two studies. Further, like the main version of the questionnaire, the effects of gender and age on the mean scores were insignificant. 

**Fig 3:**
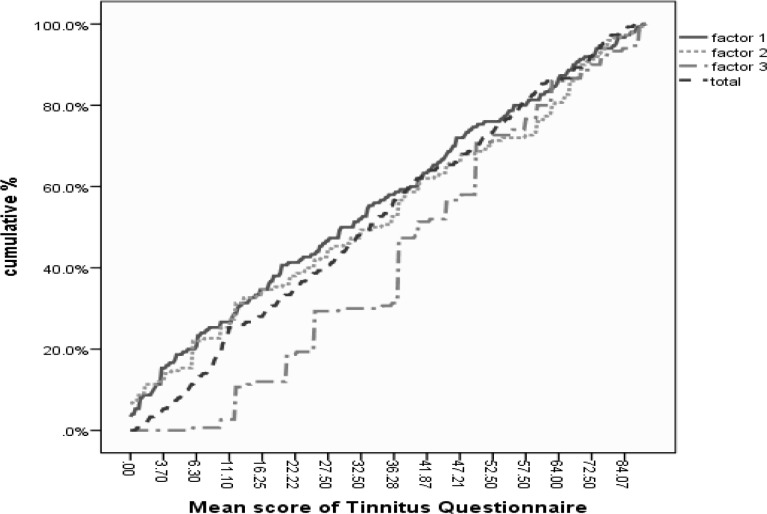
Cumulative percentage of mean score in the Persian Iowa THQ, in three factors and mean total scores (n =150)

In clinical applications, according to Kuk et al with respect to the main questionnaire, it is recommended either that factors and total score be computed in tinnitus patients, or that patients are compared with normative data. Figure 3 shows cumulative mean score in factors and total score. 

## Conclusion

In the present study, the Persian version of the Iowa THQ, administered in 150 Iranian patients suffering from tinnitus, obtained an acceptable reliability and can therefore be used in addition to other tinnitus questionnaires, as a self-report scale for objectifying the impact of tinnitus on a patient’s life and for comparing the consequences of tinnitus between patients. It also provides an effective management and treatment process across large areas of tinnitus affection, and by using wide scale (ranging from 0 to 100), allows better sensitivity and raises the possibility of detecting even small changes in the treatment and assessment process. Additionally, with regard to standardization of the questionnaire in Persian patients, it can be used in the assessment and management of Iranian patients, in addition to other questionnaires.

## References

[1] Snow JB (2004). Tinnitus: theory and management.

[2] Lockwood AH, Salvi RJ, Burkard RF (2002). Tinnitus. New England Journal of Medicine.

[3] Malouff JM, Schutte NS, Zucker LA Tinnitus-related distress: A review of recent findings. Current Psychiatry Reports.

[4] Vanneste SW, To WT, DeRidder D The psycho- metric properties of the Tinnitus Handicap Questionnaire in a Dutch Speaking population. Clinical Otolaryngology.

[5] Lewis J, Stephens S, McKenna L (1994). TINNITUS AND SUCIDE. Clinical Otolaryngology & Allied Sciences.

[6] Jastreboff PJ, Hazell JW (2004). Tinnitus retraining therapy: Implementing the neurophysiological model.

[7] Adams PF, Hendershot GE, Marano MA (1999). Current estimates from the National Health Interview Survey, 1996. Vital and health statistics.

[8] Hallam R, Jakes S, Hinchcliffe R (1988). Cognitive variables in tinnitus annoyance. British Journal of Clinical Psychology.

[9] Sweetow R, Levy M (1990). Tinnitus severity scaling for diagnostic/therapeutic usage. Hearing Instru- ments.

[10] Halford JB, Anderson SD (1991). Tinnitus severity measured by a subjective scale, audiometry and clinical judgement. J Laryngol Otol.

[11] Newman CW, Jacobson GP, Spitzer GB (1996). Development of the tinnitus handicap inventory. Archives of Otolaryngologyâ€”Head & Neck Surgery.

[12] Erlandsson SI, Hallberg LR, Axelsson A (1992). Psychological and audiological correlates of perceived tinnitus severity. International Journal of Audiology.

[13] Mahmoudian S (2011). Persian language version of the" Tinnitus Handicap Inventory": Translation, standardization, validity and reliability. International Tinnitus Journal.

[14] Kuk FK (1990). The psychometric properties of a tinnitus handicap questionnaire. Ear Hear.

[15] Meric C, Pham E, ChÃ©ry-Croze S (1997). (Translation and validation of the questionnaire" Tinnitus Handicap Questionnaire, 1990). The Journal of otolaryngology.

[16] Aaronson N (1992). International quality of life assessment (IQOLA) project. Quality of life research.

[17] Shultz KS, Whitney DJ (2004). Measurement theory in action: Case studies and exercises.

[18] Nunnally JC (1978). Psychometric Theory 3E.

[19] Newman C, Wharton J, Jacobson G (1995). Retest stability of the tinnitus handicap questionnaire. The Annals of otology, rhinology, and laryngology.

